# Successful Treatment of an Advanced Larynx Carcinoma Using Neo-Adjuvant Chemo-Immunotherapy and Cisplatin/Radiotherapy for a Patient with Rendu–Osler Disease

**DOI:** 10.3390/jcm14165694

**Published:** 2025-08-12

**Authors:** Bruno Chauffert, Tamim Alsuliman, Hanene Oueslati, Abdenour Ouikene, Farid Belkhir, Sana Nemmaoui, Alexandre Cau, Agnes Galez, Thomas Garnier, Valéry Salle, Reda Garidi

**Affiliations:** 1 Department of Oncology-Hematology, Centre Hospitalier de Saint-Quentin, 1 Av. Michel de l’Hospital BP 608, 02321 Saint-Quentin, France; tamim.alsuliman@ch-stquentin.fr (T.A.); sana.nemmaoui@ch-stquentin.fr (S.N.); 2 Department of Radiotherapy, Centre Hospitalier de Saint-Quentin, 02321 Saint-Quentin, France; 3 Department of Pharmacy, Centre Hospitalier de Saint-Quentin, 02321 Saint-Quentin, France; 4 Department of Oto-Rhino-Laryngology, Centre Hospitalier de Saint-Quentin, 02321 Saint-Quentin, France; 5 Department of Internal Medicine, CHU Amiens-Picardie, 1 Place Victor Pauchet, 80054 Amiens, France

**Keywords:** Rendu–Osler disease, squamous cell carcinoma of larynx, cisplatin-enhanced radiotherapy, chemo-immunotherapy, paclitaxel, cisplatin, cetuximab, pembrolizumab

## Abstract

**Background/Objectives**: Rendu–Osler disease is a rare genetic disease, characterized by widespread telangiectasia that can involve the skin and mucous membranes. The diagnosis is based on spontaneous and recurrent epistaxis; various mucosal and cutaneous telangiectasia at typical sites; visceral manifestations including gastrointestinal telangiectasia or pulmonary, cerebral, or hepatic arteriovenous malformation; and a first-degree relative with hereditary hemorrhagic telangiectasia. Squamous cell carcinoma of the larynx generally develops in patients with a smoking history. It is most often treated by surgery and/or radiotherapy. To our knowledge, these two entities were never reported in the same patient. **Methods:** We herein report a case of a 51-year-old man with Rendu–Osler disease. He was subsequently diagnosed with a locally advanced well-differentiated squamous cell carcinoma of the vocal cord. **Results:** The patient received a neo-adjuvant chemo-immunotherapy, with nine weekly injections of paclitaxel (60 mg/m^2^/week), cisplatin (30 mg/m^2^/week), and cetuximab (250 mg/m^2^/week), and three injections of pembrolizumab (200 mg every 3 weeks). This controlled tumor bleeding, and then cisplatin-enhanced radiotherapy helped obtain a complete remission. **Conclusions:** Locally advanced squamous cell carcinoma of the larynx treatment in the context of active Rendu–Osler disease is challenging. If the wide curative surgical approach is deemed too risky, neo-adjuvant chemo-immunotherapy may present a helpful alternative as it may enhance the conditions in order to perform classical radiotherapy with concomitant cisplatin.

## 1. Introduction

Rendu–Osler disease, also known as hereditary hemorrhagic telangiectasia (HHT), is a rare genetic disease with an autosomal dominant inheritance pattern. It is characterized by widespread telangiectasia, potentially involving the mucous membranes and the skin [1]. The dominant symptom of Rendu–Osler disease is bleeding from telangiectasia.

It can be considered a relatively rare disease in Western countries. Its prevalence varies from 1/6000 to 1/8500. No clear association with cancer was documented.

In order to facilitate the diagnosis of HHT, the Curaçao Criteria have been established. The following characteristics were considered in these criteria: recurrent and spontaneous nasal epistaxis; multiple muco-cutaneous telangiectasia; typical sites of telangiectasia; visceral manifestations including gastrointestinal telangiectasia or cerebral, hepatic, or pulmonary arteriovenous malformation; and a first-degree relative with HHT. If the case fulfills three of the aforementioned characteristic features, it can be classified as “definite”, while fulfilling only two out of them leads to marking it as “suspected”. Finally, the presence of one feature can make the diagnosis of HHT “unlikely”.

Genetic testing is not mandatory, but may confirm, if performed, the diagnosis by detecting HHT-linked pathogenic sequence variants, precisely ENG, ACVRL1, or SMAD4 [2].

Pathophysiological hypothesis corresponds to a deregulation of the angiogenic balance associated with a neo-activation of angiogenesis. The involvement of VEGF in the pathophysiology has led to the consideration of an anti-VEGF: Bevacizumab, a recombinant monoclonal antibody, inhibiting the biological activity of VEGF, whose circulating concentration is increased in Rendu–Osler disease [3]. The molecular basis can be explained by the fact that mutations in the TGF β signaling pathway may induce different phenotypes. Amongst the most concerned are endoglin (ENG in HHT1) and activin receptor-like kinase 1 (ALK1, ACVRL1 in HHT2). HHT1 is often linked to pulmonary and cerebral vascular malformations. Meanwhile, HHT2 is generally linked to vascular malformations of the liver.

This molecular hypothesis is also supported on a clinical level by the fact that vascular widening occurs in a normal capillary bed concurrently with post-capillary dilatation of venules and a decrease in the quantity of adjacent capillaries. When widening aggravates, it leads to direct shunts between arteries and veins. This can lead to telangiectasia or vascular malformations as a long-term result [1,2,3,4].

Laryngeal cancer is one of the most frequently diagnosed head and neck tumors. Squamous cell carcinoma is the most common subtype. Laryngeal cancer is most commonly detected in men aged 60 to 80 years. Its most important risk factors are alcohol and tobacco smoking. Other risk factors include environmental exposures (asbestos, textile dust, and polycyclic aromatic hydrocarbons) and dietary consumption patterns (red meat). Gastroesophageal and laryngopharyngeal reflux and HPV/p16 positivity clinical implications in laryngeal cancer pathophysiology remain unclear [5]. Laryngeal epidermoid carcinoma generally develops in smokers and is most often treated by surgery and/or radiotherapy [6]. We report a rare case of a patient with symptomatic Rendu–Osler disease and a large squamous cell carcinoma of the larynx.

## 2. Case Presentation

A 51-year-old male patient was admitted to our hospital due to abundant nasal bleeding. He had had nosebleeds since childhood. The endoglin (CD105) gene was mutated, confirming the diagnosis of a type 1 hereditary hemorrhagic telangiectasia disease (Rendu–Osler disease).

His medical history was marked by dyslipidemia treated by simvastatin, appendectomy, and dental surgeries. He also had a familial history of head and neck cancers. He was an active smoker at the time of admission. He had a history of alcohol consumption, which ceased a few years before his diagnosis with the tumor.

He received two blood transfusions during the last two years due to poorly tolerated anemia. Gastroscopy and colonoscopy were within normal limits.

**Figure 1:**
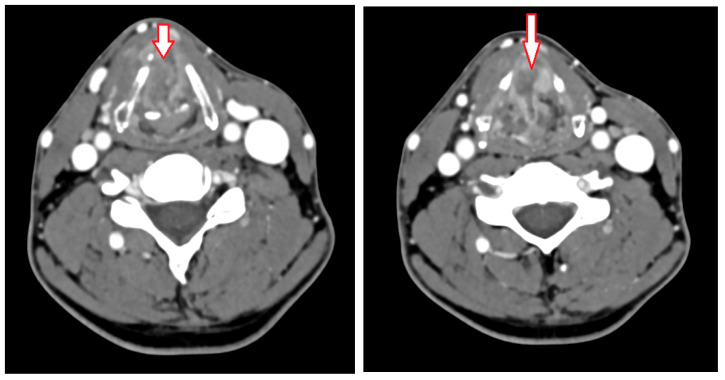
CT-scan images before the treatment showing the tumor (arrow).

He complained of dysphonia and ear pain for nearly 6 months. The first diagnosis attributed them to intubation during a recent dental surgery. A second consultation was performed due to the progression of these symptoms and to the severe progression of nasal and oral mucosal bleeding during the last month. Endoscopic examination showed a large lesion of the right hemilarynx, with glottosupraglottic and possible minor subglottic invasion. Laryngeal mobility was still preserved. A tumor of the right vocal cord with involvement of the anterior commissure was observed. Cervical computed tomography (CT), Figure 1, showed a huge necrotic mass invading the right glottic space, notably the vocal cord, with an important extension to the anterior commissure, extending beyond the midline, invading the right paralaryngeal space, and integrity of the oropharynx and hypopharynx. It also showed the mass contact with the right arytenoid, the thyroid plate on the same side, and the superior pole of the subglottic area, without clear invasion. Bilateral infra-centimetric cervical lymph nodes were also detected on CT scan.

Vocal cord biopsy was performed some days later. It was complicated with an important local and nasal hemorrhage. Histological examination indicated a differentiated squamous cell (epidermoid) carcinoma, at least microinvasive. Positron emission tomography scan showed a hypermetabolism of the right vocal cord with lysis of the thyroid cartilage. It also showed involvement signs of the cervical lymph nodes of sectors III and IV on the right side and III on the left side. There were no signs of distant metastases. The tumor was classified as T4a, N2c, and M0. Immediate surgery or radiotherapy with cisplatin was deemed unfeasible by the local head and neck cancer committee. Tracheotomy was deemed too risky given the risk of bleeding. The committee proposed a neo-adjuvant chemo-immunotherapy regimen. The patient received, during two months and under strict monitoring for bleeding, nine weekly injections of paclitaxel (60 mg/m^2^/week), cisplatin (30 mg/m^2^/week), and cetuximab (250 mg/m^2^/week), and three injections of pembrolizumab (200 mg every three weeks).

**Figure 2:**
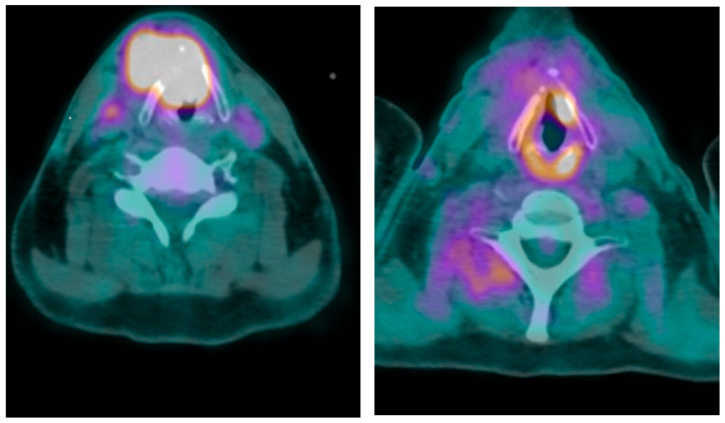
TEP-CT images showing the hypermetabolic activity of the tumor before treatment (**left**) and the metabolic response after treatment (**right**).

On post-neoadjuvant treatment evaluation, it was found that local bleeding has significantly decreased. The second phase of treatment, using radiotherapy (70 Gy, 2 Gy/day) on the tumor site and involved lymph nodes, was initiated. During the next two months, this treatment was continued in acceptable conditions with minimal local bleeding. This treatment was accompanied by concomitant cisplatin (40 mg/m^2^/week for 6 weeks). Clinical performance status and dysphonia have significantly improved at the end of treatment. Clinical examination and PET scan were normal 4 months after the end of treatment (Figure 2). The examination found Grade I-II xerostomia, for which a treatment by photobiomodulation was proposed.

**Figure 3:**
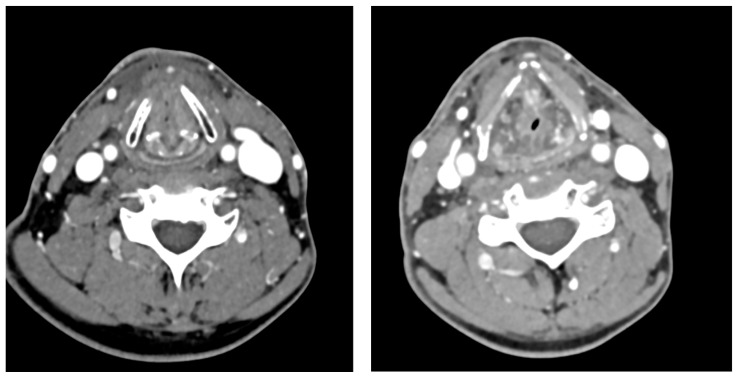
CT scan images after the treatment showing the tumor response.

At one year, cervical and thoracic CT scans were within normal limits, confirming complete remission (Figure 3). The patient had an endonasal injection of bevacizumab and cyanoacrylate glue for Rendu–Osler disease, as part of his treatment for recurrent epistaxis.

## 3. Discussion

Herein, we report a complete response of a locally advanced squamous cell carcinoma from the larynx after neo-adjuvant chemo-immunotherapy, followed by cisplatin-enhanced radiotherapy for a patient with Rendu–Osler disease. No results were found on a similar case using the PubMed database (keywords: Rendu–Osler disease and head and neck carcinoma). The initial clinical situation was challenging, due to the active bleeding from the laryngeal tumor and epistaxis. A neo-adjuvant treatment with weekly paclitaxel, cisplatin, cetuximab for 9 weeks, and pembrolizumab every 3 weeks allowed for a rapid reduction in local bleeding and laryngeal respiratory discomfort, thus avoiding the need for a tracheotomy. Curative chemo-radiotherapy with concomitant cisplatin could then be performed in a patient with a better performance status and less bleeding. Complete response was documented 12 months after starting the multi-approach treatment.

Limited laryngeal carcinoma is most often curatively managed using surgery and/or concomitant chemo-radiotherapy [7]. However, some patients presented with a bulky local/regional disease and involved cervical lymph nodes, rendering this approach very challenging.

Of note, patients with Rendu–Osler disease have high bleeding probability, either spontaneous or trauma-induced, especially from the oropharynx, nasopharynx, and digestive tube. Bleeding can also result from rupture of arteriovenous malformations in other organs and tissues. Perioperative blood loss can be massive and unpredictable in patients with this disease, as bleeding is not due to a defect in the coagulation cascade but from the exposure of malformed vascular structures resulting from the surgery [8]. Indeed, perioperative care of patients with HHT is complicated. It requires very specific interventions, especially concerning bleeding control, ensuring suitable oxygenation levels, and controlling hemodynamic values to maintain an optimal perfusion without compromising anesthetic depth [9]. These conditions deemed the wide curative surgery of our patient’s tumor so risky. Thus, our multidisciplinary committee decision was in favor of a non-surgical organ-preservative alternative approach.

Chemotherapy has been introduced into multimodality management of squamous cell carcinoma of the head and neck (SCCHN) in order to help enhance cure rates and functional results. Cisplatin was the most experimented and tested concomitant chemotherapy drug for more than half a decade, mostly because of its radio-sensitizing characteristic. In a randomized phase 3 trial in patients with high-risk profiles, Bernier et al. showed that concomitant postoperative treatment with cisplatin and radiotherapy significantly enhanced both local and regional control efficacy (hazard ratio 0.61, *p* < 0.01), with no impact on the cumulative incidence of metastases [10]. On the same issue, Cooper et al. have shown that combining cisplatin with radiotherapy has enhanced not only loco-regional control, but also progression-free survival (PFS). Both studies have administered a regimen with cisplatin at a dose of 100 mg/m^2^ every 3 weeks [11,12]. However, the TPF regimen is not always tolerated and cannot be used in unfit and frail patients [11,12].

On the other hand, the TPEX protocol, combining cisplatin, docetaxel, and cetuximab for four cycles with cetuximab maintenance, did not enhance the overall survival (OS) in advanced head and neck squamous cell carcinoma (HNSCC), but it was more tolerated with fewer toxicities than the EXTREME regimen combining cisplatin, fluorouracil, and cetuximab [13].

The KEYNOTE-048 phase 3 trial suggested that pembrolizumab, an anti-PD1 antibody, combined with a standard chemotherapy regimen containing platinum and 5-fluorouracil, is a suitable first-line treatment for Relapsed/Metastatic HNSCC [14]. A phase 2 study suggested that the association of pembrolizumab with cetuximab is an efficient and safe treatment for advanced HNSCC [15]. On this basis, we developed a tailored quadruplet regimen using paclitaxel, a platinum compound (cisplatin or carboplatin), cetuximab, and pembrolizumab to be used in some specific settings [16]. This regimen was highly active in recurrent/metastatic HNSCC. It was also active as a neo-adjuvant approach for nine patients, including two with locally advanced laryngeal tumors [17]. We have chosen the weekly version of the quadruplet in the presently frail patient. Clinical response was quickly observed, and we were able to avoid the use of a tracheotomy.

As for post-treatment supportive care, our patient received bevacizumab. In 2012, the efficacy trials of bevacizumab conducted by the Rendu–Osler Disease Reference Center in 25 patients with liver damage responsible for high-output heart failure showed encouraging results. Indeed, the literature shows that anti-angiogenic treatment based on the systemic administration of bevacizumab has demonstrated its efficacy in the treatment of arteriovenous malformations, with the observation of a simultaneous improvement in epistaxis [3].

In order to enhance our patient’s quality of life, photobiomodulation therapy (PBM-T) was proposed for the management of xerostomia. PBM-T showed efficacy in improving the quality of life of patients with head and neck cancer who received treatment with radiotherapy, alone or in combination with chemotherapy and surgery [18].

## 4. Conclusions

Locally advanced squamous cell carcinoma of the larynx treatment in the context of active Rendu–Osler disease is challenging. If the wide curative surgical approach is deemed too risky, neo-adjuvant chemo-immunotherapy may present a helpful alternative as it may enhance the conditions in order to perform a classical radiotherapy with concomitant cisplatin. Complete remission was obtained in our case, without major persistent toxicities.

## Data Availability

Data is anonymized in the manuscript. Being a case report, data is preserved and protected in the patient’s file, according to applicable laws.
